# Aetiology, prevalence, and prognosis

**DOI:** 10.1093/eurheartjsupp/suaf097

**Published:** 2025-12-26

**Authors:** Marianna Adamo, Mauro Massussi, Gianluigi Savarese, Erwan Donal, Fabien Praz, Francesco Maisano

**Affiliations:** Cardiology Unit, Department of Medical and Surgical Specialties, Radiological Sciences, and Public Health, University of Brescia, Piazzale Spedali Civili, Brescia 1–25123, Italy; Cardiac Catheterization Laboratory, Cardiothoracic Department, ASST Spedali Civili di Brescia, Piazzale Spedali CIvili 1, Brescia, Italy; Cardiac Catheterization Laboratory, Cardiothoracic Department, ASST Spedali Civili di Brescia, Piazzale Spedali CIvili 1, Brescia, Italy; Division of Cardiology, Department of Medicine, Karolinska Institute; and Heart and Vascular and Neuro Theme, Karolinska University Hospital, Stockholm, Sweden; Cardiology Unit, CHU Rennes, Inserm, LTSI—UMR 1099, University of Rennes, 2 Rue Henri le Guilloux, Rennes F-35000, France; Cardiology Unit, Inselspital Universitätsspital Bern, Bern, Switzerland; School of Medicine, Vita-Salute San Raffaele University, Milan, Italy; Department of Cardiac Surgery, IRCCS San Raffaele Hospital, Vita-Salute San Raffaele Scientific Institute, Milan, Italy

## Abstract

Tricuspid regurgitation (TR) is a common yet often overlooked valvular disorder that carries a substantial impact on morbidity and mortality. It is increasingly recognized as a heterogeneous entity with different phenotypes identified (primary, atrial secondary, ventricular secondary, and cardiac implantable electronic device-related). Contemporary population studies and disease-specific registries reveal that secondary TR is highly prevalent in elderly patients, those with heart failure of any phenotype, and in candidates for transcatheter aortic or mitral interventions. Prognosis varies widely according to aetiology, with atrial secondary TR consistently associated with better survival than ventricular secondary TR. Across diverse settings, TR severity is an independent predictor of mortality, and several clinical scores, including the TRI-SCORE, Wang score, and TRIO score, have been developed to refine risk stratification. Recent staging models integrating ventricular function, renal status, and biomarkers suggest that intervention during an intermediate disease phase, before irreversible end-organ damage, may optimize outcomes. Together, these advances underscore the need for accurate phenotyping, structured prognostic assessment, and timely intervention to improve the care of patients with TR.

## Introduction

Tricuspid regurgitation (TR) is a frequently encountered valvular abnormality, often identified incidentally during routine echocardiographic assessment.^1^ Moderate or severe TR affects approximately 4% of individuals over the age of 75 years—an incidence comparable to that of clinically relevant aortic stenosis or mitral regurgitation.^2^

Despite its prevalence, TR has long been under-recognized and under-treated. Historically regarded as a benign consequence of left-sided heart disease or pulmonary diseases, it was assumed that TR would improve with treatment of the primary pathology.^2,3^ This perception contributed to a delay in the development of diagnostic and therapeutic strategies targeting TR specifically. Furthermore, when TR is not associated to left-sided heart disease, symptoms are often aspecific at early stages. Thus, TR is not recognized and patients are not referred to specific management.^4^

However, several studies have demonstrated that moderate-to-severe TR is independently associated with poor clinical outcomes, including significantly increased mortality—even after adjustment for comorbid conditions.^5^ Sex-based differences have emerged, with women disproportionately affected by significant TR. Female sex has been identified as an independent predictor of disease severity and progression, possibly related to a higher prevalence of atrial fibrillation (AF) and heart failure with preserved ejection fraction (HFpEF).^6^ Additional predictors of TR progression include advancing age, pulmonary hypertension (PH), and atrial remodeling.

In light of growing awareness, mainly due to availability of new strategies for treatment, TR is increasingly being recognized as a disease entity with distinct pathophysiological subtypes, a relevant epidemiological burden, and important prognostic implications. This review addresses three key aspects: aetiological classification of TR, current estimates of prevalence, and the evolving understanding of its prognosis and natural history.

## Classification and aetiology of tricuspid regurgitation

TR is broadly classified into primary TR (pTR), secondary TR (sTR) and cardiac implantable electronic device (CIED) related, based on the underlying mechanism of valve dysfunction—a distinction with important prognostic and therapeutic implications.^7^

pTR, accounting for approximately 5–10% of cases, results from intrinsic abnormalities of the tricuspid valve apparatus, such as leaflet prolapse, congenital malformations, rheumatic disease, infective endocarditis, carcinoid syndrome, or infiltrative disorders.^8,9^ Iatrogenic causes, such as repeated endomyocardial biopsies in heart transplant recipients, also fall into this category.^10^ In pTR, valve incompetence is due to structural damage, typically without significant changes in right heart geometry, at least at the initial stages.

By contrast, sTR—which accounts for the vast majority of cases (80%)—is a functional abnormality of structurally normal leaflets, caused by right heart chamber remodeling. Secondary TR arises from annular dilatation and leaflet tethering due to primary or secondary PH leading to right ventricular (RV) overload (i.e. left-sided heart disease or pulmonary disease), or primary RV (i.e. cardiomyopathy, myocardial infarction), or atrial dysfunction (i.e. long-standing AF).^11^

Contemporary classifications further stratify sTR into two distinct phenotypes with differing pathophysiology and prognostic profiles: ventricular sTR (V-sTR) and atrial sTR (A-sTR).

**Figure 1:**
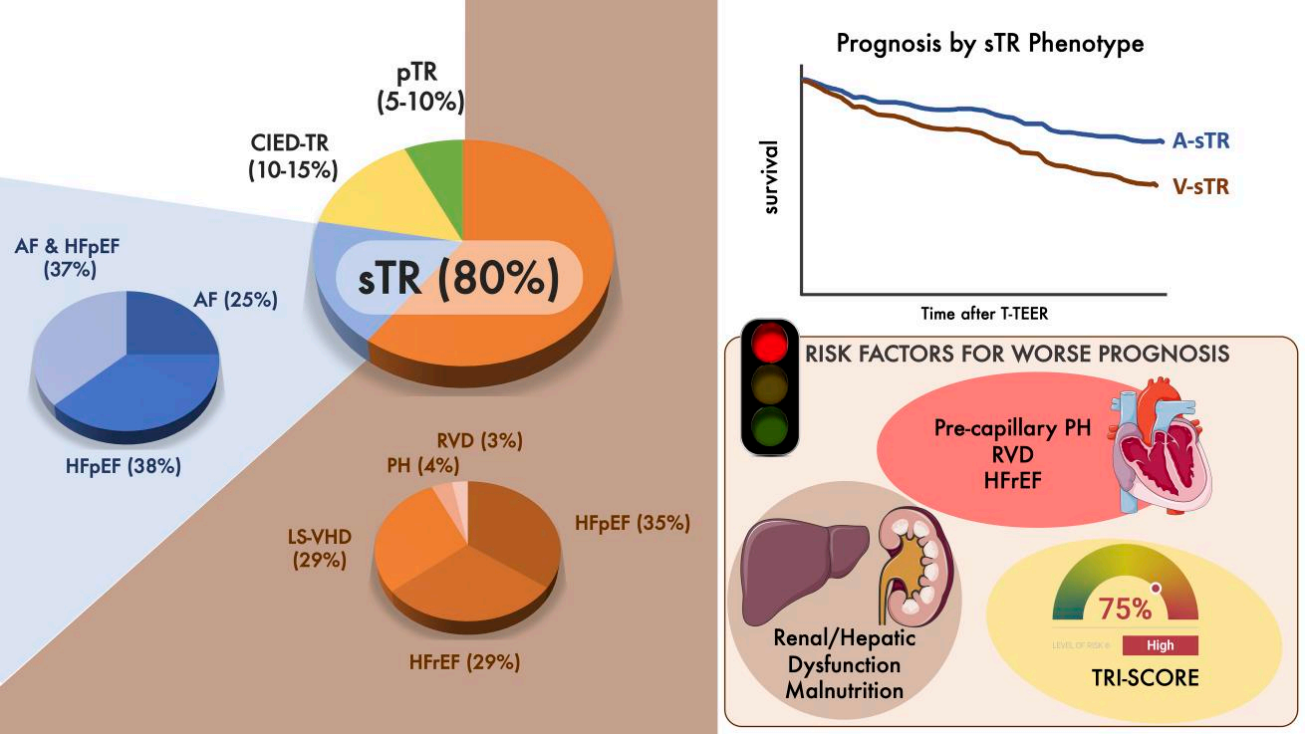
Prevalence of TR based on aetiology and prognostic aspects. pTR, primary tricuspid regurgitation; sTR, secondary tricuspid regurgitation; CIED-TR, cardiac implantable electronic device-related tricuspid regurgitation; A-sTR, atrial secondary tricuspid regurgitation; V-sTR, ventricular secondary tricuspid regurgitation; AF, atrial fibrillation; HFpEF, heart failure with preserved ejection fraction; HFrEF, heart failure with reduced ejection fraction; LS-VHD, left-sided valvular heart disease; PH, pulmonary hypertension; RVD, right ventricular dysfunction; T-TEER, transcatheter tricuspid edge-to-edge repair.

V-sTR results from RV pressure, leading to RV remodeling, papillary muscle displacement, and leaflet tethering. Common underlying conditions include left-sided HF, PH, and intrinsic myocardial disease. The CARE-TR registry proposed a refined subclassification of V-sTR into five subgroups:^12^ severe left-sided valvular heart disease (LS-VHD); heart failure with reduced ejection fraction (HFrEF) or mildly reduced ejection fraction (HFmrEF) without severe LS-VHD; HFpEF without severe LS-VHD; pre-capillary PH; isolated RV dysfunction (RVD) without left ventricular disease (*Figure 1*).

This heterogeneity within V-sTR highlights the need for tailored management strategies.

Atrial sTR, a recently recognized phenotype, is associated with permanent AF and age-related atrial remodeling. It is characterized by isolated annular dilatation due to right atrial enlargement, typically occurring without RVD, PH, or significant left-sided heart disease. Although often less symptomatic at onset, A-sTR can progress and is associated with an increased risk of adverse outcomes, particularly in patients with persistent AF and HFpEF.^12^ A recent expert opinion paper defines A-sTR, its risk factors and possible overlap conditions.^13^

A third emerging phenotype is CIED-related TR, which has become increasingly common with the widespread use of pacemakers and defibrillators.^14^ CIED-related TR can result from direct mechanical interference of the lead with leaflet motion or from pacing-induced RVD (dyssynchrony) and annular dilatation; both mechanisms often coexist. This form of TR affects up to 25–45% of device recipients and is associated with rapid progression, higher hospitalization rates, and increased mortality.

This nuanced classification—encompassing PTR, V-sTR, A-sTR, and CIED-related TR—provides a foundation for individualized diagnosis, risk stratification, and emerging targeted therapies.^15^

## Epidemiology of tricuspid regurgitation

### Prevalence of tricuspid regurgitation in heart failure

Secondary TR is highly prevalent among patients with HF, affecting individuals across the spectrum of HF phenotypes. Moderate-to-severe TR has been reported in approximately 10–23% of HF patients in previous observational studies.^1,16,17^ In the ESC-HF Long-Term Registry, moderate-to-severe TR was present in 19.2% of the cohort, with similar rates across HF subtypes (19% in HFrEF, 17.8% in HFmrEF, and 20.3% in HFpEF)^18^ and with isolated TR being more frequently observed in HFrEF as compared to TR combined with moderate-to-severe mitral regurgitation.^19^ Heitzinger et al. showed a prevalence of severe TR higher in HFrEF as compared to HFmrEF and HFpEF (20% vs. 10% and 9%), although the absolute number of patients with severe TR was higher in HFpEF than other HF phenotypes since the latter were less represented.^20^

### Prevalence of tricuspid regurgitation in patients undergoing transcatheter aortic and mitral valve interventions

TR is also common among patients undergoing transcatheter interventions for LS-VHD, particularly transcatheter aortic valve implantation (TAVI) and mitral transcatheter edge-to-edge repair (M-TEER). In the TVT registry, moderate TR was present in 13.1% and severe TR in 2.3% of TAVI patients, while in the M-TEER group, 33.2% had moderate TR and 14.7% had severe TR.^21^ Similar data were observed in other European studies showing a prevalence of severe TR in 2.8% of TAVI candidates^22^ and 27% of M-TEER recipients.^23^ These findings highlight the frequent coexistence of TR in elderly patients with left-sided valvular disease, further complicating management and outcomes.

### Prevalence of tricuspid regurgitation subtypes in contemporary registries

Recent registries have provided important insights into the distribution and clinical characteristics of TR subtypes, including A-sTR, and V-sTR.

The CARE-TR registry, a multicenter Italian cohort of 648 patients with severe or greater sTR, reported A-sTR in 22.1% and V-sTR in 77.9% of cases.^12^ A-sTR was strongly associated with AF and HFpEF, either alone or in combination, and was more frequent among women, who tended to have fewer comorbidities and milder symptoms compared to patients with V-sTR. In contrast, V-sTR was more often linked to left-sided valvular disease, PH, and myocardial dysfunction, and was characterized by more advanced structural and haemodynamic abnormalities.

Additional observational studies, including patients conservatively managed, have reported A-sTR prevalence ranging between 13% and 26%, depending on the population studied and the criteria used to define A-sTR.^13,24-26^

The TRIVALVE registry, which included 298 patients undergoing transcatheter tricuspid valve repair (TTVR), similarly reported an A-sTR prevalence of 22%, with the remaining 78% having V-sTR. Patients with V-sTR exhibited more pronounced haemodynamic compromise, higher NT-proBNP levels, and greater symptom burden than those with A-sTR, underscoring the distinct pathophysiological and clinical profiles of these subtypes.^27^

A combined analysis of the Leiden and Leipzig cohorts—including both conservatively managed patients and those undergoing TTVR^26^ proposed an echocardiographic definition of A-sTR, derived using a bioinformatic approach. This definition identifies approximately 15% of patients with significant TR as having a likely underlying atrial mechanism.

These findings collectively highlight the increasing recognition of A-sTR as a distinct phenotype, particularly in elderly patients with AF and preserved LV and RV function.

In the EuroTR registry, including patients undergoing transcatheter tricuspid valve edge-to-edge repair (T-TEER), further illustrated the heterogeneity of TR aetiology. In this cohort, TR was classified as primary in 7.6%, secondary in 84.1%, and mixed primary/secondary in 8.3%. A sub-analysis showed a prevalence of A-sTR of 31%.^28^ Notably, 28.2% of patients had a *trans*-tricuspid pacing lead, highlighting the substantial burden of CIED-related TR in contemporary practice. The majority of patients in this cohort presented with HFpEF (72%), with smaller proportions having HFmrEF (16.5%) or HFrEF (11.4%).^29^

Together, these findings underscore the heterogeneity of TR in terms of aetiology and clinical presentation and emphasize the importance of distinguishing among its subtypes.

## Prognostic role of tricuspid regurgitation

### Clinical progression and risk stratification

TR is probably not merely a marker of advanced disease but a possible active contributor to morbidity and mortality, owing to its haemodynamic effects including systemic congestion and decrease in forward stroke volume leading to multi-organ dysfunction. Indeed, TR-related congestion contributes to cardiorenal syndrome (impaired renal function), cardio-hepatic syndrome (liver injury with cholestatic pattern and reduced synthetic function), and gastrointestinal dysfunction (malabsorption, malnutrition, increased intestinal permeability), forming a broader multi-organ syndrome.^30-32^

Importantly, TR may progress insidiously, with mild and nonspecific symptoms such as peripheral oedema and fatigue, underscoring the need for early recognition and intervention.^4^

Identifying patients with worse prognosis who could benefit from closer monitoring and timely intervention remains a critical unmet need. In this context, several risk stratification tools have been proposed to predict mortality in patients with significant TR (*Table 1*). The TRI-SCORE^33^ was developed to estimate operative mortality in patients undergoing isolated tricuspid valve surgery and it was validated in different settings^34,35^; the TRIVALVE score was derived to assess prognosis after TTVI^36^; the Wang score was designed to predict 1-year mortality risk in patients with isolated TR managed medically;^37^ and the TRIO score was introduced to stratify mortality risk in patients with notable TR across broader clinical settings.^38^ All these scores have demonstrated prognostic value for mortality prediction at follow-up. However, each has limitations: their derivation cohorts were highly selected, only one has been externally validated,^39^ comparative performance remains unknown, and their ability to predict broader clinical outcomes beyond mortality has yet to be established.

**Table 1:** Risk stratification tools for tricuspid regurgitation

	TRI score	TRIO score	Wang score	TRIVALVE Score
**Parameters**	Age ≥70 years (+1) NYHA functional Class III–IV(+1) Right-sided heart failure signs (+2) Daily furosemide ≥125 mg (+2) Glomerular filtration rate <30 mL/min (+2) Elevated total bilirubin (+2) Left ventricular ejection fraction <60% (+1) TAPSE <17 mm or S'DTI <9.5 cm/s, or visual RV dysfunction (+1)	Age 70–79 years (+1); ≥ 80 years (+2) Male (+1) Chronic lung disease (+1) Congestive HF (+1) Creatinine ≥2 mg/dL (+2) AST ≥40 U/L (+1) HR ≥90 beats/min (+1) Severe TR (+1)	Age 65–74 years (+1); ≥ 75 years (+2) Myocardial Infarction (+1) Chronic lung disease (+1) Loop diuretic use (+1) PAD (+1) Hb < 10 g/dL (+1) INR >1.5 (+1) Creatinine ≥1.4 mg/dL (+1) Albumin <3.0 g/dL (+2) Platelets < 15 000/mL (+1) RV SP >50 mmHg (+1) RV dysfunction (mild +1, moderate +2, severe +3)	Atrial Fibrillation (+1) Glomerular filtration rate <30 mL/min (+1) Elevated GGT or Bilirubin (+1) Signs of right HF (+1) Left ventricular ejection fraction <50% (+0.5)
**Maximum score**	Total: 12 points	Total: 12 points	Total 16 points	Total: 4.5 points
	**High severity score: ≥ 10 points**	**High severity score: ≥ 7 points**	**High severity score: ≥ 12 points**	**High severity score: ≥ 2.5 points**
**Purpose**	Mortality risk in isolated TV surgery	Long-term mortality risk in patients with significant TR	1-year mortality in patients with isolated TR	Mortality/HF hospitalization in patients undergoing transcatheter tricuspid valve intervention
**Validation**	**Widely externally validated (multiple independent cohorts across Europe and internationally)**	**Externally validated (geographically distinct cohort, and tested in independent cohorts)**	**Partially validated externally (tested in independent cohorts in comparative studies, but no dedicated external validation paper)**	**Not externally validated** **(only internally/derivation validation so far)**

### Prognostic role of tricuspid regurgitation in heart failure

Secondary TR is associated with significantly worse survival in patients with HF, regardless of HF phenotype and setting (chronic, acute, advanced).^17,19,20,40-42^ The Vienna HF-TR study provided a comprehensive analysis of sTR’s prognostic impact across the full HF spectrum. In this longitudinal cohort of 13 469 HF patients without primary valvular disease, even moderate sTR was associated with substantial excess mortality compared to patients with no or mild sTR, and this risk increased further with severe sTR.^20^ During a median follow-up of 44 months, 27.3% of patients died. At 4 years, the mortality rate for patients with severe sTR was 44%, compared with 24% for those with no/mild sTR and only 2% in the age- and sex-matched general population. At 8 years, these rates increased to 61%, 38%, and 15%, respectively. Adjusted HR confirmed that moderate sTR was associated with a 58% increase in mortality risk (HR 1.58, 95% CI 1.48–1.69), while severe sTR conferred more than a twofold increase (HR 2.19, 95% CI 2.01–2.38). These associations remained significant after adjustment for clinical and bootstrap confounder models. Subgroup analyses revealed that the negative impact of sTR was particularly pronounced in younger patients (<70 years: HR 1.97 vs. ≥ 70 years: HR 1.66, *P* for interaction = 0.040), as well as in those with hypertension, ischaemic heart disease, larger LV dimensions, and preserved RV function. Interestingly, in patients with severely impaired RV function, the association between sTR severity and mortality was attenuated, highlighting the complex interplay between RV failure and TR.^20^ Complementary insights from the ESC-HFA HF long-term registry further highlight the prognostic relevance of TR in HF.^19^ Among 11 298 HF outpatients with no significant aortic disease and over a median follow-up of 12 months, patients with isolated TR and those with combined MR/TR had markedly higher rates of all-cause mortality (12.8 and 13.3 events/100 patient-years, respectively) compared to patients with isolated MR (9.9/100 patient-years, respectively) or no significant valve disease (5.0/100 patient-years, respectively). After adjustment, isolated TR and combined MR/TR were independently associated with worse survival (HR 1.42 and 1.45, respectively), and similar trends were observed for cardiovascular death and HF hospitalization. Both isolated TR and combined MR/TR were independently associated with higher mortality and HF hospitalization compared to isolated MR or no valve disease, underscoring the particularly adverse prognosis conferred by TR in HF, especially in HFpEF. Data from Mayo Clinic on 13 026 patients with HFrEF, showed that, as compared to trivial TR, higher TR grades were independently associated with higher mortality (adjusted hazard ratios 1.09 [1.01–1.17] for mild TR, 1.21 [1.11–1.33] for moderate TR and 1.57 [1.39–1.78] for severe TR).^17^ Finally, the GALACTIC-HF trial, which enrolled over 8000 patients with symptomatic HFrEF, confirmed that moderate-to-severe TR was independently associated with worse outcomes, even after adjustment for clinical covariates. Patients with moderate/severe TR had a significantly higher risk of the composite endpoint of HF events or CV death compared to those with no or mild TR (adjusted HR 1.12–1.14), primarily driven by an increased incidence of HF events and hospitalizations.^40^

Taken together, these findings emphasize that sTR may not be merely a bystander but a key contributor to adverse outcomes in HF, across phenotypes and severities, warranting earlier recognition and targeted intervention.

### Prognostic role of tricuspid regurgitation in transcatheter valve populations

The large analysis from the TVT registry showed that baseline moderate-to-severe TR was associated with significantly higher mortality and readmission rates at both 30 days and 1 year after TAVI and M-TEER. After multivariable adjustment, patients undergoing TAVI with moderate TR had a 24% increased risk of 1-year mortality (HR 1.24), and those with severe TR had a 65% increase (HR 1.65) compared with patients with none or mild TR. Similarly, among M-TEER recipients, moderate TR was associated with a 17% higher 1-year mortality (HR 1.17), and severe TR with a 49% higher risk (HR 1.49). One-year readmission rates and health status, as measured by the Kansas City Cardiomyopathy Questionnaire (KCCQ), were also significantly worse in patients with moderate or severe TR, who achieved smaller improvements in quality of life despite intervention.^21^

A *post hoc* analysis of the COAPT trial suggested that M-TEER may mitigate the poor prognosis associated with TR in patients with secondary mitral regurgitation, though these findings are exploratory.^43^ Together, these observations underscore the importance of assessing TR severity and considering its management in patients undergoing transcatheter left-sided interventions. Notably, severe TR was an exclusion criterion in the COAPT randomized trial, limiting conclusions about the efficacy of M-TEER in this subgroup. However, a large multicenter European registry specifically examined the evolution of TR after M-TEER and its impact on long-term outcomes.^23^ Among 503 patients with secondary MR undergoing successful M-TEER, TR improved by at least one grade in ∼35%, remained stable in ∼46%, and worsened in ∼19% at short-term follow-up (median ∼3 months). Improvement was more frequent in patients with higher baseline TR (≥2+) and was independently associated with smaller left atrial diameter and optimal MR reduction. Importantly, the degree of TR at short-term follow-up—rather than at baseline—was a strong predictor of long-term mortality: patients with TR ≤2 + had a 42% lower risk of death compared to those with TR ≥3+ (HR 0.58, *P* = 0.011), regardless of baseline TR. These findings support a strategy of reassessment of TR after M-TEER and consideration of staged tricuspid intervention if significant TR persists.

### Prognosis according to secondary tricuspid regurgitation phenotype

Recent evidence suggests that outcomes in sTR vary substantially according to its underlying phenotype, with A-sTR consistently associated with a more favourable prognosis compared with V-sTR or other non-atrial forms.^25^

The prognostic heterogeneity of sTR phenotypes has been demonstrated in several large cohorts of patients managed both medically and transcatheter. The CARE-TR registry (*n* = 648) provided detailed phenotypic and outcome data in patients with severe sTR, showing that at 2 years, survival free from the composite of death or HF hospitalization was significantly higher in patients with A-sTR compared with those with V-sTR (73.5% vs. 54.4%, *P* < 0.001), with V-sTR independently associated with a higher risk (adjusted HR 2.00). Within A-sTR, outcomes were worst in patients with both AF and HFpEF compared to AF or HFpEF alone, while within V-sTR, the poorest outcomes were seen in patients with pre-capillary PH and severe LS-VHD.^12^

These findings were documented also by the Leiden cohort (*n* = 1037), which applied a structured classification of sTR into atrial and ventricular phenotypes. Over a median follow-up of 78 months, patients with A-sTR had markedly better survival compared to V-sTR (10-year survival: 78% vs. 46%, *P* < 0.001), with V-sTR independently associated with worse outcomes (HR ∼2.3). Among V-sTR subtypes, survival was worst in those with PH and RVD, whereas A-sTR consistently showed the most favourable prognosis.^24^

Finally, the large Cleveland Clinic series by Wang et al. (*n* = 9045) further underscored the survival advantage of atrial over ventricular-driven forms of sTR. In this cohort, unadjusted 5-year survival was significantly better in atrial functional TR compared to sTR from left-sided disease or PH. In addition, Wang et al. developed and validated a risk prediction model (Wang score) for sTR, incorporating clinical, echocardiographic, and laboratory markers of RVD, PH, renal and hepatic impairment, and comorbidities. This score demonstrated good discrimination (C-statistic ∼0.73) and provides a practical tool to estimate 1-year mortality and guide management decisions.^37^

Consistent findings have also emerged from cohorts of patients undergoing TTVR and T-TEER, reinforcing the prognostic advantage of A-sTR over V-sTR. In the TRIVALVE registry A-sTR was associated with significantly better survival compared with V-sTR at a median follow-up of 10 months (91% vs. 72%, *P* = 0.02). Multivariate analysis identified procedural success as an independent protective factor for mortality (HR 0.41), while V-sTR and high-dose diuretic use showed a trend toward worse outcomes.^27^ The EuroTR registry, including 641 patients treated with T-TEER, confirmed these findings. One- and two-year survival rates were significantly higher in patients with A-sTR than in those with non-atrial sTR (1 year: 84.0% vs. 77.6%; 2 years: 79.3% vs. 63.8%; both *P* < 0.001). Similarly, survival free from HF hospitalization at 1 and 2 years favoured A-sTR over non-atrial sTR. In multivariable Cox regression, A-sTR was independently associated with improved two-year survival free from heart failure hospitalization (HR 0.65, *P* = 0.025).^28^

Taken together, these data consistently support that A-sTR represents a more favourable phenotype, even in medically treated patients, and highlight the importance of structured risk stratification to optimize care (*Figure 1*).

### Prognosis according to stage of disease

Several markers of advanced HF (i.e. recurrent HF hospitalizations, malnutrition, RV dysfunction, PH, clinical signs of right HF) have been reported as associated with poor outcome in patients with TR, especially in the setting of TTVI.^44-48^

A large multicenter analysis of 1885 patients with severe symptomatic TR by Schlotter et al. proposed a disease stage classification based on a multiparameter score integrating biventricular function, renal dysfunction, and neurohormonal activation (BNP/NT-proBNP) derived from patients undergoing T-TEER.^49^ Patients were categorized into early, intermediate, and advanced stages, which correlated with progressively higher 1-year mortality (6%, 15%, and 31%, respectively; *P* < 0.01). When comparing T-TEER to conservative therapy across stages, T-TEER was associated with significantly lower 1-year mortality in the intermediate stage (13% vs. 21%; HR 0.73; *P* = 0.03), but no benefit was observed in early or advanced stages. These findings suggest that intervention at the intermediate stage—before irreversible end-organ damage and haemodynamic collapse—may optimize outcomes.

A sub-analysis of the TRIVALVE cohort specifically examined patients with RVD and PH. Among 300 patients meeting these criteria, RVD was present in 81%, PH in 42%, and both in 24%. Prognosis in this high-risk group was strongly influenced by end-organ damage and procedural success. Patients with significant renal dysfunction (eGFR <45 mL/min) and/or hepatic congestion had markedly higher estimated 1-year mortality rates (44%), rising to 58% when procedural failure was also present. Notably, patients with neither renal/hepatic dysfunction nor procedural failure demonstrated survival comparable to those with procedural success despite some end-organ impairment. These findings underscore the critical impact of right-sided end-organ dysfunction and procedural success on outcomes in patients with advanced TR undergoing transcatheter therapy.^50^

## Conclusion

TR is a prevalent and clinically significant valvular disorder, most commonly sTR in nature, with distinct phenotypes including ventricular and atrial forms. TR is reported as a possible independent driver of morbidity and mortality, since it may contribute to a multi-organ syndrome. Contemporary registries have highlighted its high prevalence, particularly in elderly patients, those with heart failure, as well as those undergoing transcatheter interventions for left-sided valve disease or CIED.

The heterogeneous pathophysiology of TR and its substantial prognostic role underscore the need for improved awareness, early diagnosis, and timely intervention before irreversible right heart and end-organ dysfunction develop.

## Data Availability

Not applicable.
